# Inhibitors of Acetylcholinesterase and Butyrylcholinesterase Meet Immunity

**DOI:** 10.3390/ijms15069809

**Published:** 2014-06-02

**Authors:** Miroslav Pohanka

**Affiliations:** 1 Faculty of Military Health Sciences, University of Defence, Trebesska 1575, Hradec Kralove CZ-50001, Czech Republic; E-Mail: miroslav.pohanka@gmail.com; Tel./Fax: +420-973-253-091; 2 Karel English College in Brno, Sujanovo namesti 356/1, Brno 60200, Czech Republic

**Keywords:** cholinergic anti-inflammatory pathway, acetylcholinesterase, butyrylcholinesterase, Alzheimer disease, neuroimmunity, neuromodulation

## Abstract

Acetylcholinesterase (AChE) inhibitors are widely used for the symptomatic treatment of Alzheimer’s disease and other dementias. More recent use is for myasthenia gravis. Many of these inhibitors interact with the second known cholinesterase, butyrylcholinesterase (BChE). Further, evidence shows that acetylcholine plays a role in suppression of cytokine release through a “cholinergic anti-inflammatory pathway” which raises questions about the role of these inhibitors in the immune system. This review covers research and discussion of the role of the inhibitors in modulating the immune response using as examples the commonly available drugs, donepezil, galantamine, huperzine, neostigmine and pyridostigmine. Major attention is given to the cholinergic anti-inflammatory pathway, a well-described link between the central nervous system and terminal effector cells in the immune system.

## 1. Introduction

Inhibitors of AChE (EC 3.1.1.7.) and BChE (EC 3.1.1.8) are neurotoxic compounds capable of causing central, peripheral or both central and peripheral cholinergic crises. A number of these compounds have also found application as drugs developed for the treatment of Alzheimer’s disease (AD) and myasthenia gravis [1,2]. These are based on the premise that increasing the availability of acetylcholine (ACh) at acetylcholine receptors in the brain, results in better neuron to neuron transport that will improve cognitive function. Cholinergic nerves, however, can be found in both the central (CNS) and peripheral (PNS) nervous systems and disparate body tissues [3]. Drugs that cross the blood brain barrier do not have dissociable groups as can be seen in the case of AD drugs [4]. Some of these drugs cannot penetrate the CNS and this property makes them suitable for use in myasthenia gravis [5]. For a long time, regulation of immunity was not considered an effect of AChE inhibitors. However, recent evidence casts new light on the subject. In this review, we explore the link between immunity and the AChE inhibitors as currently available AChE inhibiting drugs for AD.

## 2. The Cholinergic Anti-Inflammatory Pathway

Ach is a ubiquitous neurotransmitter [6,7] and found even in the roundworm *Caenorhabditis elegans*, one of the simplest organisms with a nervous system [8,9]. In the roundworm one third of the nervous system is cholinergic [10]. Humans have a large percentage of nervous system that is cholinergic including the CNS. Cholinergic nerves also form a major part of the parasympathetic and sympathetic nervous systems [11,12]. The wider significance of Ach is in understanding the biological effects of tested toxins and/or medical drugs: as any immunological effects of AChE inhibitors can involve both CNS and PNS, this has to be taken into consideration in interpreting any findings. For this reason, vagotomy, used to study the cholinergic anti-inflammatory pathway in animal experiments or selecting compounds that do not cross the blood brain barrier should be considered carefully before drawing any conclusions as to which pathway is involved in the proposed mechanism. 

The cholinergic system is tightly associated with the cholinergic anti-inflammatory pathway dominantly located in blood and mucosa. This pathway is a regulatory link between nerve terminations in blood and macrophages expressing the α7 nicotinic acetylcholine receptor (α7 nAChR) on their surface [11,13,14]. For a long time, the mechanisms of inflammatory regulation remained unclear. Discovery of the cholinergic anti-inflammatory pathway, however, allowed us to understand how the CNS is involved in the regulation of innate immunity [15,16,17,18,19,20,21,22,23,24]. AChE bound on erythrocytes plays an important role in termination of cholinergic anti-inflammatory pathway activation [11,25]. AChE activity is typically low in AD patients treated with AChE inhibitors [26]. Compared to AChE, BChE is constituted in the liver and secreted into the plasma where the enzyme is dissolved [27]. Apart from the fact that the conversion rate of Ach by BChE is lower than the conversion by AChE, BChE can substitute for AChE and split the neurotransmitter once they make contact [28,29]. The effect of BChE became relevant once the cholinergic anti-inflammatory pathway was studied as BChE plays a greater role in the blood than in the nervous system. 

**Figure 1:**
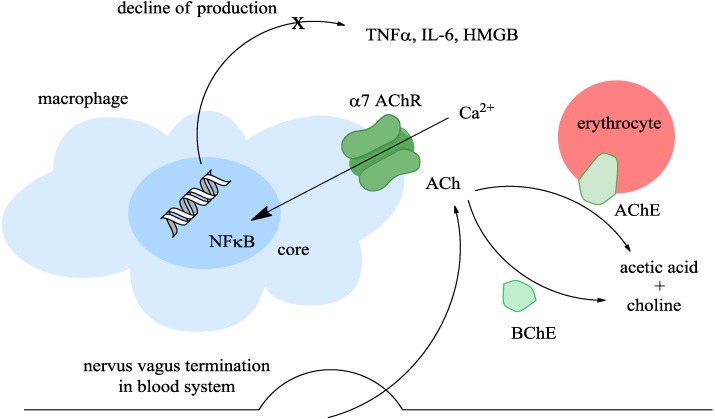
Principle of the cholinergic anti-inflammatory pathway; abbreviations: ACh-acetylcholine; AChE-acetylcholinesterase; BChE-butyrylcholinesterase; HMGB-high mobility group box; IL-6-interleukin 6; NFκB-nuclear factor kappa B; TNFα-tumor necrosis factor alpha.

The cholinergic anti-inflammatory pathway is one-way: the CNS can attenuate inflammation mediated by macrophages or any other immune cells having α7 nAChR. Ach released from the vagus nerve termination, agonizes α7 nAChR, which responds by opening a central channel allowing an influx of Ca^2+^ into macrophages [11,30,31]. Increased levels of Ca^2+^ activate the nuclear factor κ B (NF κB) resulting in suppression of inflammatory cytokine production including tumor necrosis factor α (TNFα), high mobility group box of proteins and interleukin 6 (IL-6) [32,33]. These blood AChE and plasma BChE are able to terminate the stimulation of the cholinergic anti-inflammatory pathway due to splitting ACh. The principle of the pathway is depicted in Figure 1.

The primary purpose of using AChE inhibitors in pharmacology is not modulation of immunity related pathologies. However, recent studies indicate that these inhibitors can cause a significant modulation of immunity as a side effect [29,34,35]. As seen from Figure 1, they can modulate the cholinergic anti-inflammatory pathway via protection of Ach from splitting by cholinesterases and thus enhancing the pathway. The mechanism of action is probably less effective than the standard mode but it becomes relevant when someone is using an inhibitor of cholinesterases in large amounts and/or for a long time such as patients suffering from AD.

Apart from the regulation processes, some inhibitors can influence immunity via forming antigens by reaction with e.g., plasma proteins. The immune system is thus activated and the stimulation counteracts the anti-inflammatory action. This effect is, however, very weak but it can play a role in forming antibody proteins modified by nerve agents [36].

## 3. Division of Inhibitors

The structure of AChE and BChE has been extensively reviewed in the following publications [7,29,37,38]. In brief, AChE has a more developed peripheral anionic site and narrower aromatic gorge than BChE. Aromatic compounds have higher affinity for AChE than BChE. Some aromatic inhibitors of AChE do not inhibit BChE, for example aflatoxins [39,40]. When the role of the cholinesterases is evaluated in humans, their genomic diversity and posttranslational modifications have to be taken into account [41,42].

Organophosphorus compounds are irreversible inhibitors of both AChE and BChE. They bind to the active site of the cholinesterases and easily cross the blood brain barrier [6,43]. Nerve agents, e.g., sarin, soman, tabun, and some highly toxic compounds, formerly used as pesticides (paraoxon, parathion, malaoxon, malathion), are examples [44,45,46,47]. High toxicity characterizes organophosphorus inhibitors that are used in chemical warfare or as pesticides. Their pharmacological importance is relatively small; Metrifonate (trichlorfon) was chosen for AD treatment and became an exception but it was withdrawn because of adverse effects [48,49,50]. Though organophosphorus compounds typically inhibit both AChE and BChE, tetraisopropyl pyrophosphoramide, also known as iso-OMPA, is dissimilar to the other inhibitors. It does not penetrate to the active site of AChE and it inhibits BChE only. It is typically used as a reagent for rapid distinction between AChE and BChE activity in biological samples [51,52].

Carbamate inhibitors bind to the active site of both cholinesterases like organophosphorus inhibitors; however, the covalent bound is not stable and the carbamate moiety is hydrolytically split from the active site after some time [29,53,54]. The mechanism of carbamate binding is sometimes called pseudo-irreversible because of carbamate moiety spontaneous hydrolysis and resurrection of cholinesterase activity. From a pharmacological point of view, there is a big difference between carbamates and organophosphorus inhibitors. Many carbamates do not cross the blood brain barrier and the carbamate moiety has to be modified or encapsulated to cross it [55,56,57]. Quarternary ammonium containing pyridostigmine and neostigmine are examples. On the other hand, the blood brain barrier is not impenetrable by all carbamates e.g., rivastigmine (see further text) and physostigmine easily reach AChE in the brain [58,59,60]. 

AChE and BChE can be inhibited by a group of secondary metabolites from plants and fungi. Galantamine and huperzine are examples of plant alkaloids used in pharmacology. Alkaloids α-chaconine, α-solanine, tomatine, berberine, palmatine and jatrorrhizine are other metabolites that inhibit cholinesterases [29,61,62,63,64,65]. Aflatoxins too, can be introduced as secondary metabolites from fungi. 

Disparate synthetic drugs not belonging to carbamates and organophosphates can be mentioned last but not least. Donepezil is the most relevant compound of this group. (See the next chapter). Tacrine (1,2,3,4-tetrahydroacridin-9-amine) is another synthetic drug which easily crosses the blood brain barrier and is used as a highly effective drug for ameliorating Alzheimer disease manifestation by inhibition AChE and by lower but still effective inhibition of BChE [53,66,67]. It was withdrawn from clinical use because of adverse effects. Hepatotoxicity was the main pathological consequence of tacrine intake [68,69]. The basic facts about groups of inhibitors are summarized in Table 1.

In considering the role of the inhibitors in modulating immunity, other factors such as the environment and genetic disposition should be taken into account. This conclusion is based on blood cholinesterase activity in male volunteers working with pesticides [70]. The activity varied in infected patients with proven bacterial meningitis [71]. The sensitivity of humans to the inhibitors can also significantly be affected because detoxification mechanisms have unequal efficacy. This idea was, e.g., demonstrated by Sonali *et al*.on AD patients treated with rivastigmine [72]. Variability in inhibitor effects should be considered when conclusions are drawn from animal models and cell lines, extrapolated to humans. 

**Table 1:** Summarization of facts about cholinesterases’ inhibitors.

Group of Compounds	Compounds (Examples)	Mechanism of Inhibition	Inhibition of AChE and BChE	Penetration through Blood Brain Barrier	Importance as Drugs	References
Organophosphates	sarin, soman, tabun, malaoxon	irreversible	equal to AChE and BChE	Good	Low	[6,43,44,45,46,47,48,49,50]
Carbamates	pyridostigmine, physostigmine neostigmine, rivastigmine	pseudo-irreversible	equal to AChE and BChE	low (pyridostigmine, neostigmine), good (physostigmine, rivastigmine)	High	[29,53,54,55,56,57,58,59,60]
-	Tacrine	non-competitive	AChE > BChE	Good	former drug, discontinued now	[53,66,68,69]
-	Galantamine	competitive	AChE	Good	High	[73,74]
-	Donepezil	non-competitive	AChE	Good	High	[75,76]
-	huperzine A	non-competitive	AChE >> BChE	Good	will increase	[29,75,77,78,79]

## 4. Inhibitors that Cross the Blood Brain Barrier

**Figure 2:**
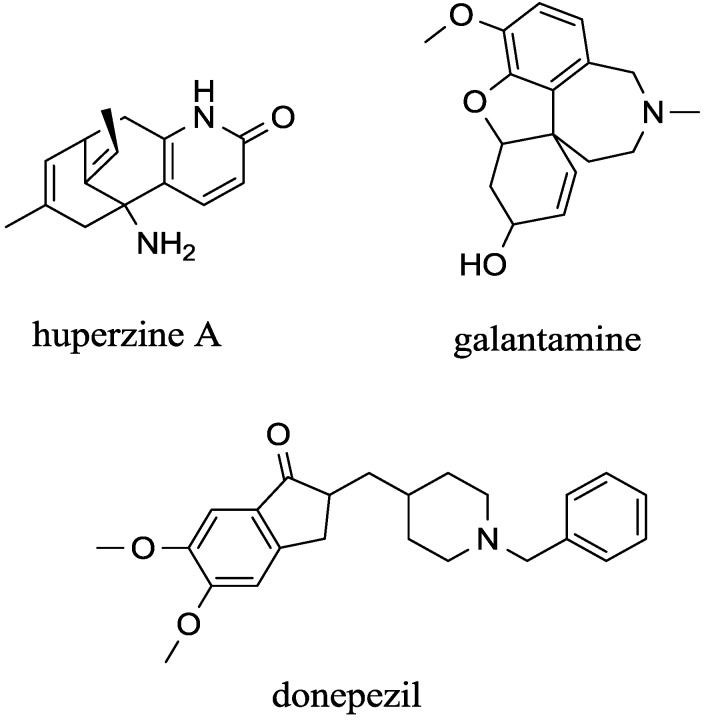
Structure of cited compounds that cross the blood brain barrier.

The following text focuses on galantamine, donepezil, huperzine and rivastigmine. Galantamine (Figure 2) is a drug used for treating AD and related dementias. Currently, galantamine is an alternative to rivastigmine and can be given to patients with similar stages of dementia. On the market, it is sold under the trade names Razadyne™, Razadyne™ER, Reminyl™ER, and Reminyl^®^ The drug was firstly isolated by soviet scientists Mashkovsky and Kruglikova-Lvova from bulbs of Caucasian snowdrops *Galanthus* sp. in the early 1950s and chemical synthesis was introduced in the following decades [80]. After marketing of the drug, it drove out the more toxic tacrine [81] and has become one of the best drugs for Alzheimer disease treatment [73].

Galantamine is a competitive inhibitor of AChE and an allosteric modulator of nAChR [74]. Its dual action on both AChE and nAChR is an advantage and unlike other marketed drugs that inhibit AChE. It is believed that galantamine may interact with the cholinergic anti-inflammatory pathway via direct modulation of the α7 nAChR [82]. The anti-inflammatory pathway modulation may explain the activation of microglia followed by amyloid beta clearance [83]. In a rat model, galantamine was also approved as effective in reducing circulating TNFα which was, in the past, initiated by administration of bacterial lipopolysaccharide [84]. For this reason, galantamine could act not only as a drug for symptomatic treatment but as a compound for slowing Alzheimer disease progression. This conclusion is not, however, commonly accepted and more detailed evidence of the process is needed. 

**Figure 3:**
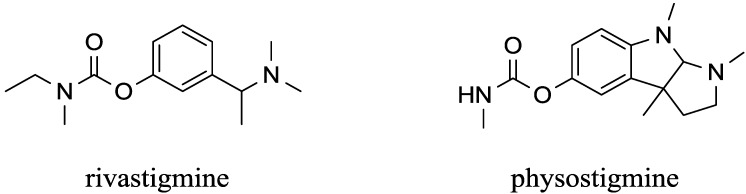
Structure of carbamates that cross the blood brain barrier.

Donepezil is a selective, noncompetitive inhibitor of AChE. The inhibition is quite effective as the equilibrium constant is reported to be 12.5 nmol/L for AChE from rat erythrocytes [75]. Donepezil is available under the trade name Aricept as a highly effective drug for Alzheimer disease, originally developed by Eisai and Pfizer. The structure of donepezil is depicted in Figure 3. Clinical experience with donepezil is good: it is well tolerated and slowly eliminated so that the drug can be taken over long periods [76]. Compared to other drugs for Alzheimer disease, donepezil works via a simple pathway based on AChE inhibition. It is not involved in other pathways and does not involve BChE inhibition [29].

We can assume that donepezil activates the cholinergic anti-inflammatory pathway via inhibition of AChE and increased availability of ACh. However, Hwang and coworkers found anti-inflammatory effects on microglia cell lines where no AChE was present [85]. These authors showed significant attenuation of TNFα, IL-1, and NF-κB release. From these results, we can infer that donepezil is either able to directly stimulate α7 nAChR and not act as an inhibitor only or it has some other unknown pathway. Beside the anti-inflammatory pathway, donepezil was proven to be able to modulate viral progression and the modulation is probably based on a mechanism other than agonism of α7 nAChR [86,87]. This fact would support the results on the microglia cells. Unfortunately, the antiviral effect of donepezil was not studied further, though the issue deserves greater attention.

Huperzine is a secondary metabolite from the lycopods, *Huperzia* from which it is isolated for pharmacological purposes. The upper production of huperzine alkaloids can be found in *H*. *serata*. More types of huperzine are known. However, huperzine A (Figure 2) is the most likely to be used in Alzeheimer disease [75]. Elaborative isolation of huperzine from plant biomass is the main disadvantage of huperzine as chemical synthesis is problematic due to expensive isolation of the (−) huperzine A from the (+) isomer which is not pharmacologically relevant [88]. Though advanced chemical synthesis protocols [89] and biotechnology processes [90] for the (−) huperzine A production are described, they are far from practical use. Huperzine is used in traditional Chinese medicine and it is available as a supplement in the country. It can be taken as a mechanically milled plant tissue or as an extract prepared by chromatographic isolation [77]. In countries of the European Union and United States, pro-drug ZT-1 derived from huperzine A is clinically tested and introduction for therapy purposes is expected in the near future [91]. 

Huperzine A is a selective inhibitor of AChE, acting by a non-competitive or mixed mechanism [75]. Huperzine A can bind to the peripheral anionic site of AChE and the effect is reportedly responsible for amelioration of the amyloidogenic process [92]. Besides AChE inhibition, huperzine A is a potent non-competitive inhibitor of the *N*-methyl-d-aspartate receptor [78,79].

Huperzine A was proven to reduce neuroinflammation in experimental autoimmune encephalomyelitis in mice [93]. The authors reported a decrease in the number of inflammatory cells, interferon gamma (IFNγ), IL-17, MCP-1, RANTES, TWEAK and an increase in IL-4 and IL-10 in the course of treatment. In another experiment, huperzine inhibited activation of NF-κB, inducible nitric oxide synthase and cyclooxygenase 2 in C6 rat glioma cells [94]. Though direct molecular evidence is missing, agonism of α7 nAChR with subsequent activation of the cholinergic anti-inflammatory pathway is presumed to be involved in huperzine’s mechanism of action [95].

Rivastigmine (Figure 3) is a carbamate inhibitor of AChE as well as BChE and it easily crosses the blood brain barrier. Rivastigmine is probably the most marketed carbamate in pharmacology. The drug is sold under the trade name Exelon for the treatment of Alzheimer and Parkinson disease in early and mild stages. Currently, it is the only available drug for these diseases, which is not a reversible inhibitor of AChE since it inhibits the cholinesterases in a pseudo-irreversible manner [96]. Slow elimination of rivastigmine because of the covalent bond in the active site of the enzyme is an advantage over other drugs. The effect of rivastigmine can last until the rivastigmine moiety spontaneously splits from the cholinesterase’s active site by a decarbonylation process [4,54]. 

The long-term effects of rivastigmine remain unclear. It was proven that rivastigmine causes significant up-regulation of AChE expression [97]. The molecular mechanism is, however, unrevealed. In clinical tests, rivastigmine was not found to generate inflammation or have any other adverse effect. For this reason, rivastigmine is considered as a quite safe drug [98]. On the contrary, more detailed examination showed that rivastigmine can suppress inflammation [99]. Namely, decreased reactivity of encephalitogenic T lymphocytes and production of pro-inflammatory cytokines was reported [100]. More experiments on this issue will be necessary as the exact mechanism is not clear. It can be assumed that the cholinergic anti-inflammatory pathway can be activated as Ach becomes available for the α7 nAChR on macrophages and microglial cells. Direct proof is, however, missing. 

Like rivastigmine, physostigmine (or eserine in some sources) crosses the blood brain barrier and can inhibit AChE and BChE in the both central and peripheral nervous systems. Physostigmine is a carbamate of natural origin that can be found in the seeds from a plant *Physostigma venosum* known as the Calabar bean. At the current time, cheap and reliable protocols for physostigmine synthesis are available and preferred over isolation from plants [101]. Physostigmine can be used for alleviation of glaucoma manifestation [102] and it is suitable for the treatment of Alzheimer disease [103,104]. As discussed later in the text, physostigmine can inhibit protein kinase C. This ability is not common to other carbamate inhibitors of cholinesterases [105]. Physostigmine was shown to increase the availability of ACh and stimulate the cholinergic anti-inflammatory pathway in experimental endotoxemia by lipopolysaccharide [106]. In another experiment, physostigmine regulated early inflammation and oxidative stress as the superoxide radical in rats with induced forebrain ischemia [107].

## 5. Peripherally Acting Carbamates-Parasympathomimetics

**Figure 4:**
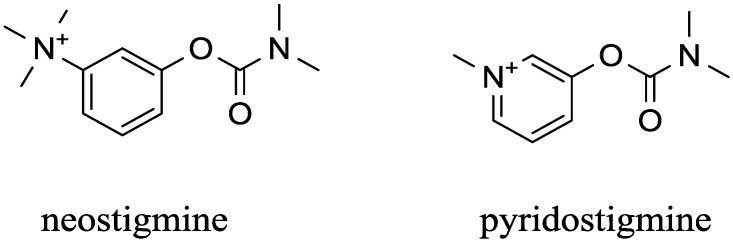
Structure of carbamate inhibitors that do not cross the blood brain barrier.

Most carbamates have limited ability to pass the blood brain barrier. Rivastigmine is an exception from this point of view. The poor ability to cross the blood brain barrier can be an advantage when we need a compound to regulate the PNS and not the CNS. Drugs that do this are used for two main purposes: in the treatment of myasthenia gravis [108] and in anesthesia [109]. Myasthenia gravis is an autoimmune disease where antibodies against acetylcholine receptors are created in the body. The treatment the disease can be based on administration of immunosuppressant and/or an AChE inhibitor [110]. In surgical interventions, there is a necessity to give muscle relaxants such as parasympathomimetics. The parasympathomimetics can act as polarizing agents via direct stimulation of receptors (e.g., succinylcholine) or as non-polarizing agents where the peripherally acting carbamates belong [111,112]. Pyridostigmine and neostigmine are well known examples of carbamates acting as parasympathomimetics [113]. Beside the carbamates, a quaternary nitrogen containing inhibitor edrophonium is also used as a parasympathomimetic [114]. The chemical structures of neostigmine and pyridostigmine are shown in Figure 4. Though the blood brain barrier seems to be impenetrable for compounds like pyridostigmine and neostigmine, the contrary is true. For example, Friedman *et al*. showed that stress conditions can cause penetrability of blood brain barrier by pyridostigmine [115]. This factor has to be considered in critical evaluation of peripherally acting carbamates. Strong stressogenic conditions can cause peripherally acting carbamates to have the same effect as a centrally acting one. 

The peripherally acting carbamates have similar action to rivastigmine. They are pseudo-irreversible inhibitors of both AChE and BChE [29]. Selective effect on the PNS is the main difference between the carbamates such as neostigmine and pyridostigmine and the centrally acting rivastigmine. The inability to cross blood brain barrier discriminates the peripherally acting carbamates from being used for regulation of neuroinflammation and other immunity related disorders within the CNS. On the other hand, they can be expected to be favored in the generalized immunity disorder regulation whenever brain functions should be spared.

Plausible anti-inflammatory effects of the peripherally acting carbamates have been described in some papers. In an example, physostigmine (crossing blood brain barrier) and neostigmine (not crossing) reduced inflammation which was previously initiated by a bacterial lipopolysaccharide [116,117]. Similar results were reported in an experiment where mice were infected with tularemia and treated with neostigmine [118]. In this experiment, neostigmine worsened the tularemia pathology. Sun *et al*.tested neostigmine effects in mice and Beagle dogs [119]. They showed that neostigmine acts via the cholinergic anti-inflammatory pathway. This conclusion is supported by the fact that mice with knockout gene for α7 nAChR are not sensitive to the immunomodulatory effect. On the other hand, more experiments on the issue will be needed as some did not confirm any significant anti-inflammatory effect of carbamates [120]. Regulation of immunity can be based on pathways far from the cholinergic system. e.g., Bitzinger *et al.* revealed the ability of physostigmine to inhibit protein kinase C while neostigmine did not have this ability [105]. 

## 6. Conclusions

**Figure 5:**
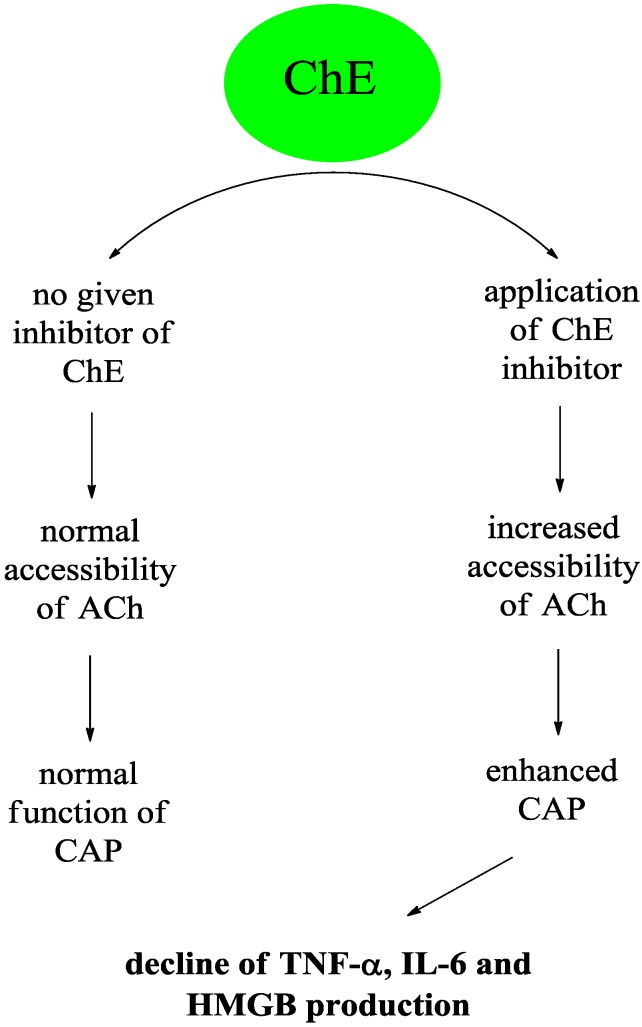
Simplified scheme for showing how inhibitors of cholinesterases may be involved in regulation of immunity using the cholinergic anti-inflammatory pathway. Abbreviations used in the figure: ACh—acetylcholine; CAP—cholinergic anti-inflammatory pathway; ChE—cholinesterase; HMGB—high mobility group box proteins.

Currently available drugs for inhibiting either AChE alone or in combination with BChE are available for the treatment of AD, myasthenia gravis and other conditions. These drugs however, are not used for immunomodulation purposes at this time. This review highlights the fact that these inhibitors may affect not only the cholinergic anti-inflammatory pathway but also other unknown pathways involved in regulating immunity. A simplified mechanism for how these inhibitors may be involved in regulating immunity is depicted schematically in Figure 5. This issue deserves greater attention due to its pharmacological relevance.
